# Genetic landscape features associated with PD-L1 expression status in advanced lung adenocarcinoma: a targeted sequencing analysis

**DOI:** 10.3389/fonc.2026.1892351

**Published:** 2026-07-23

**Authors:** Mukun Huang, Zhongqiang Yao, Hua Zhang, Chuanjun Song, Mengzhu Wang, Jiangman Zhao, Shushen Ji, Keliang Zhu, Mengxin Zhao, Dan Zhang

**Affiliations:** 1 Department of Pharmacy, 3201 Hospital, Hanzhong, Shaanxi, China; 2 Department of Medical Oncology, 3201 Hospital, Hanzhong, Shaanxi, China; 3 Department of Thoracic Surgery, Shandong Public Health Clinical Center, Jinan, Shandong, China; 4 Department of Oncology, Xinghua People’s Hospital, Taizhou, Jiangsu, China; 5 Shanghai Biotecan Medical Laboratory Co., Ltd., Shanghai Zhangjiang Institute of Medical Innovation, Shanghai, China; 6 Department of Medical Oncology II, The Second Affiliated Hospital of Xi ‘an Medical University, Xi ‘an, Shaanxi, China

**Keywords:** genomic landscape, lung adenocarcinoma, mutation burden, pathway enrichment, PD-L1

## Abstract

**Background:**

The tumor immune microenvironment influences non-small cell lung cancer (NSCLC) progression and therapy response. Programmed death-ligand 1 (PD-L1) is a key biomarker for immune checkpoint blockade, yet the genomic correlates of its expression status require elucidation.

**Methods:**

We analyzed 97 advanced lung adenocarcinoma patients, (13 cases—all from the PD-L1-unknown subgroup—excluded due to insufficient sequencing quality, leaving 84 cases for downstream analyses) categorized by PD-L1 expression (positive: n=32; negative: n=45; unknown: n=20). Genomic profiling was performed using a 95-gene targeted NGS panel. Analyses included mutational profiling, pathway enrichment (GO/KEGG), gene interaction patterns, and genomic complexity.

**Results:**

The cohort was predominantly metastatic (99.0%). Dominant mutations were in EGFR(43.80%), TP53(38.02%), and KRAS(11.57%). PD-L1-positive tumors more frequently harbored EGFRE746_A750del, while TP53 mutations were enriched in PD-L1-negative cases. Somatic interaction analysis revealed significant EGFR/TP53 co-occurrence in PD-L1-positive tumors and TP53/RB1 co-occurrence in negative tumors. GO analysis showed PD-L1-positive tumors were enriched for kinase-related functions, reflecting a distinct EGFR-driven biology characterized by a striking 59.26% prevalence of EGFR mutations. KEGG analysis indicated immune pathway enrichment in this subgroup, supporting an oncogene-induced immune phenotype frequently synergized by TP53 co-alterations. In contrast, PD-L1-negative tumors correlated with co-mutation-prone, metabolism-oriented profiles and immune suppression. Mutual exclusivity patterns differed: in PD-L1-positive tumors, EGFRmutations were mutually exclusive with KRAS/NRAS/HRASmutations, suggesting a single dominant driver. PD-L1-negative tumors displayed more co-occurring alterations (e.g., TP53 with STK11 or PTEN). Furthermore, 24% of PD-L1-positive tumors had multiple concurrent alterations, a proportion significantly higher than in PD-L1-negative tumors (8%; P<0.05).

**Conclusion:**

This study delineates distinct genomic landscapes associated with PD-L1 expression. PD-L1-positive status is linked to an immune-active, kinase-signaling-driven phenotype with mutually exclusive drivers. PD-L1-negative status correlates with a more immunosuppressive, co-mutation-prone, and metabolism-oriented profile. These findings provide molecular insights into immune heterogeneity and may inform tailored combination therapies.

## Introduction

Lung cancer persists as the leading cause of cancer-related mortality globally, with non-small cell lung cancer (NSCLC) constituting approximately 85% of all cases (1, 2). Despite progress in diagnosis and treatment, the prognosis for patients with advanced or metastatic NSCLC remains poor, historically associated with a 5-year overall survival rate below 5% (3). The emergence of targeted therapies and immune checkpoint inhibitors (ICIs) has transformed the therapeutic landscape, markedly improving outcomes for defined molecular subgroups (4, 5). Notably, ICIs targeting the programmed death-1 (PD-1)/programmed death-ligand 1 (PD-L1) axis have shown significant efficacy in a subset of NSCLC patients (6). Currently, PD-L1 protein expression on tumor cells, as evaluated by immunohistochemistry (IHC), is the most widely adopted predictive biomarker for anti-PD-1/PD-L1 therapy (7). However, its predictive value is not absolute, as responses occur in some patients with low or negative PD-L1 expression, while others with high expression may not benefit (8, 9). This highlights the complexity and heterogeneity of the tumor immune microenvironment, indicating that PD-L1 expression alone is an insufficient proxy for the intricate interplay between tumors and the host immune system.

The genomic landscape of NSCLC is marked by considerable heterogeneity, driven by somatic mutations, copy number alterations, and gene fusions (10, 11). Seminal studies have identified recurrent driver alterations in genes such as EGFR, KRAS, ALK, ROS1, BRAF, and MET, which have enabled genotype-directed therapies (12, 13). Beyond these established drivers, a tumor’s mutational profile can substantially influence its immunogenicity and the composition of the tumor microenvironment (TME) (14). For instance, a high tumor mutational burden (TMB) has been linked to increased neoantigen load and improved responses to Immune Checkpoint Inhibitors (ICIs) in several cancers, including NSCLC (15, 16). Additionally, specific mutational signatures and co-alterations may modulate immune evasion and therapeutic resistance (17). The TP53tumor suppressor gene is the most frequently mutated gene in NSCLC (18). Emerging evidence suggests that TP53 mutations are not only crucial in tumorigenesis but also correlate with a more aggressive phenotype and may influence the immune contexture (19). Notably, co-occurring TP53mutations have been investigated as potential poor prognostic factors in patients receiving targeted therapies like EGFR-tyrosine kinase inhibitors (TKIs) (20, 21), and their role in shaping immunotherapy response is an active area of research.

Technological advances, particularly next-generation sequencing (NGS), have enabled comprehensive genomic profiling, providing unprecedented insights into cancer biology (22). While tumor tissue DNA sequencing remains the gold standard, its application can be limited by tumor inaccessibility, insufficient material, or intra-tumoral heterogeneity (23). Liquid biopsy, specifically the analysis of circulating tumor DNA (ctDNA), offers a promising, non-invasive alternative for capturing tumor-derived genomic alterations (24, 25). Studies indicate that although ctDNA detection sensitivity can be lower than tissue-based assays, the mutational landscape derived from plasma is largely concordant with tissue profiles, especially in lung adenocarcinoma (LUAD) (26). This supports the utility of ctDNA for genomic subtyping, monitoring clonal evolution, and identifying actionable alterations to guide clinical management (27, 28). The feasibility and clinical actionability of genomic profiling—from tissue or plasma—are well-established, with a significant proportion of NSCLC patients harboring at least one actionable genomic alteration according to databases like OncoKB (29).

Metastasis, particularly to sites such as bone, is a major cause of morbidity and mortality in NSCLC, severely worsening patient prognosis (30). The molecular determinants predisposing tumors to metastasize to specific organs are not fully understood. Comparative genomic studies between metastatic and non-metastatic cohorts have begun to uncover distinct genetic features associated with metastatic potential (31). For example, differences in somatic mutation spectra, co-occurring and mutually exclusive gene pairs, and enriched signaling pathways have been reported between advanced NSCLC patients with and without bone metastasis (32). Furthermore, systemic factors, including the accumulation of specific heavy metals in serum, have been explored for their potential association with metastasis and may interact with genomic alterations (33). Understanding these distinct biological characteristics is vital for identifying high-risk patients and developing novel strategies to prevent or treat metastasis.

Beyond genomic and metastatic profiling, the molecular heterogeneity of NSCLC, particularly LUAD, necessitates refined classification systems to advance precision medicine. Multi-omics approaches integrating transcriptomic and proteomic data have successfully identified novel molecular subtypes of lung adenocarcinoma with distinct biological traits and clinical behaviors (34). Such subtypes, driven by mechanisms like EIF4G1-mediated proliferation, KRAS activation, or core serum response signatures, may not be fully explained by single-gene driver mutations and offer new avenues for subtype-specific interventions (35–40). This underscores the need to move beyond a single-biomarker paradigm toward a more integrated molecular understanding of the disease.

Within this context, the precise relationship between the baseline tumor genomic architecture and PD-L1-mediated immune status in NSCLC warrants deeper exploration. The genetic correlates underlying the variable expression and functional state of PD-L1 are not fully delineated. A systematic comparison of the mutational landscape across patients stratified by PD-L1 protein expression status (positive, negative, and untested) could reveal genetic features that cooperate with or regulate the PD-1/PD-L1 axis. Therefore, this study aims to investigate the genetic landscape features associated with immune status in NSCLC. We performed targeted NGS on a cohort of 97 tumor tissue samples from NSCLC patients, categorized into PD-L1-negative (n=45), PD-L1-positive (n=32), and PD-L1-unknown (n=20) groups. Our primary objectives are to: (1) delineate and compare the hotspot gene mutation spectra and frequencies among these groups; (2) analyze these genomic alterations in the context of key clinical variables including gender, smoking history, histology, and tumor differentiation; (3) identify potential co-alteration patterns and enriched biological pathways that distinguish different immune statuses. By integrating genomic data with immune marker status, this research seeks to uncover the molecular underpinnings of immune heterogeneity in NSCLC, which may contribute to developing more effective biomarkers and combination therapeutic strategies.

## Materials and methods

### Study cohort and patient characteristics

A total of 97 patients with histologically confirmed lung adenocarcinoma were enrolled in this study between May 2023 and December 2025 at the Department of Oncology II, Hanzhong 3201 Hospital. All patients met the following inclusion criteria: (1) age ≥ 18 years; (2) first diagnosis of lung adenocarcinoma confirmed by histopathology according to the 4th edition of the World Health Organization Classification of Lung Tumors; (3) available formalin-fixed, paraffin-embedded (FFPE) tumor tissue samples for genomic analysis; (4) available PD-L1 expression data determined by immunohistochemistry (IHC); (5) no prior anticancer treatment (including chemotherapy, radiotherapy, targeted therapy, or immunotherapy) before sample collection. Patients with other concomitant malignancies, autoimmune diseases, or active infections requiring systemic therapy were excluded. The study was approved by the Institutional Review Board of Hanzhong 3201 Hospital (approval number: LL-QT-2025-026-01), and written informed consent was obtained from all participants in accordance with the Declaration of Helsinki.

### PD-L1 expression assessment and patient stratification

PD-L1 expression was evaluated using IHC on FFPE tumor sections with the commercially available 22C3 pharmDx assay (Agilent Technologies, Santa Clara, CA, USA) or an equivalent validated assay. PD-L1 expression was quantified as the tumor proportion score (TPS), defined as the percentage of viable tumor cells showing partial or complete membranous staining. According to routine clinical cutoffs, a TPS ≥ 1% was considered PD-L1 positive, TPS < 1% as PD-L1 negative. Cases with insufficient tumor content or technical failure in IHC assessment were recorded as PD-L1 expression unknown. Based on this, patients were stratified into three groups for subsequent comparative genomic analyses: PD-L1 positive (n=32), PD-L1 negative (n=45), and PD-L1 unknown (n=20). All comparative analyses were performed between the PD-L1–positive (n=32) and PD-L1–negative (n=45) subgroups; the PD-L1–unknown cohort (n=20) was excluded. Certain sub-analyses involving specific driver gene panels were performed on subsets of these cohorts with adequate data completeness for the genes of interest.

### DNA extraction, library preparation, and targeted sequencing

Genomic DNA (gDNA) was extracted from FFPE tumor tissue specimens using the GeneRead DNA FFPE Kit (Qiagen GmbH, Hilden, Germany) according to the manufacturer’s instructions. DNA quantity, purity, and integrity were rigorously assessed. Only samples with sufficient DNA yield (≥100 ng) and passing quality control (average yield ratio ≥0.3 *via* a multiplex PCR-based GAPDH fragment assay) were processed. The “average yield ratio” refers to a quantitative metric derived from a multiplex PCR-based assay targeting the GAPDH(glyceraldehyde-3-phosphate dehydrogenase) gene.

For each qualified sample, 300 ng of gDNA was fragmented, and sequencing libraries were constructed using the KAPA Hyper Prep Kit (Kapa Biosystems). Libraries were then subjected to hybridization capture using a custom-designed xGen Lockdown Probe pool (Integrated DNA Technologies) targeting 95 cancer-related genes. The captured libraries were sequenced on an Illumina NextSeq CN500 platform, generating 150 bp paired-end reads.

### Bioinformatic analysis of genomic alterations

Raw sequencing data were processed through a standard bioinformatics pipeline. This included adapter trimming, quality filtering, alignment to the human reference genome (hg19/GRCh37) using BWA-MEM, duplicate read marking, and local realignment/base quality score recalibration using the Genome Analysis Toolkit (GATK). Somatic single nucleotide variants (SNVs) and small insertions/deletions (indels) were called using VarDict, utilizing matched peripheral blood leukocytes or adjacent normal tissue as a control to distinguish somatic from germline variants.​ In the absence of a patient-matched normal sample for a subset of cases, we relied on stringent population frequency filtering to minimize the inclusion of common germline polymorphisms.

The called variants were annotated using ANNOVAR and then underwent a series of filters to select high-confidence somatic alterations. Filtering criteria included: (i) minimum sequencing depth of 10x; (ii) low allele frequency in population databases (MAF ≤ 0.001 in 1000 Genomes/ExAC East Asian cohorts), with an exception for variants listed in the COSMIC database; (iii) location within exonic or splice site regions; (iv) exclusion of synonymous variants; and (v) exclusion of variants predicted to be benign by in silico tools (PolyPhen-2, MutationTaster). Copy number variations (CNVs) were detected using CNVkit with the hg19/GRCh37 reference. A pooled set of normal samples from the cohort was used to generate a reference for segmentation and copy number ratio calculation, as patient-matched normal tissue was not available for all cases.

### Functional enrichment and somatic interaction analysis

To explore the biological implications of the genomic alterations, we performed functional enrichment analysis. Gene Ontology (GO) biological process and Kyoto Encyclopedia of Genes and Genomes (KEGG) pathway enrichment analyses were conducted on the lists of genes harboring somatic mutations within each PD-L1 subgroup, using the clusterProfiler R package. Terms with a Benjamini-Hochberg adjusted P-value < 0.05 were considered significantly enriched.

To decipher patterns of genetic interaction, co-occurrence and mutual exclusivity analyses were performed. For this analysis, we focused on a predefined set of core driver and significantly mutated genes, which included all genes from the targeted panel that were mutated in at least 10% of the samples within either the PD-L1-positive or PD-L1-negative cohort. Pairwise interaction analyses for these genes were conducted separately within each subgroup using Fisher’s exact test. Given the exploratory nature of this analysis, a nominal P-value < 0.1 was used as the threshold to identify suggestive co-occurrence or mutual exclusivity patterns. To account for multiple testing, the Benjamini–Hochberg false discovery rate (FDR) correction was applied.

### Annotation of genomic alterations for clinical actionability

The potential clinical relevance of all identified somatic mutations was evaluated using the OncoKB Precision Oncology Database (accessed May 2025). Each alteration was classified into one of four levels according to OncoKB evidence: Level 1 (FDA-recognized predictive biomarker), Level 2 (standard care biomarker per guidelines), Level 3 (actionable in clinical trials), or Level 4 (biologically relevant with preclinical evidence).

### Statistical analysis

All statistical analyses were performed using R software and GraphPad Prism. Clinical and genomic characteristics were compared between the PD-L1-positive and PD-L1-negative groups (the PD-L1-unknown cohort was excluded from these comparative analyses). Continuous variables, such as patient age, tumor mutation frequency, and variant allele frequencies, were assessed for normality and compared using either the Student’s t-test (for normally distributed data) or the non-parametric Mann-Whitney U test. Categorical variables, including clinical-pathological parameters (e.g., sex, smoking status, disease stage) and the mutation status of individual genes, were compared using the Chi-square test or Fisher’s exact test, as appropriate. All statistical tests were two-sided. All statistical tests were two-sided. A two-sided P<0.05 was considered statistically significant for all confirmatory comparative analyses (e.g., clinical characteristics, mutation frequencies, genomic complexity), while a nominal P<0.1 was reserved exclusively for the exploratory gene–gene interaction analyses to identify suggestive patterns, consistent with the hypothesis-generating nature of these specific analyses.

## Results

### Patient characteristics

A total of 97 patients with lung adenocarcinoma were enrolled in this study, including 32 patients with PD−L1 positive expression (33.0%), 45 with PD−L1 negative expression (46.4%), and 20 with unknown PD−L1 status (20.6%). The demographic and clinical characteristics of the patients are summarized in **Table 1**. No significant differences in age (mean 65.60 ± 10.36 years), gender (male 52.6%, female 47.4%), smoking history (non−smoker 61.9%), or BMI (mean 21.68 ± 2.85) were observed between the PD−L1 positive and negative groups (all P > 0.05). The majority of tumors were adenocarcinoma (93.8%), with moderate differentiation accounting for 71.1%. Notably, the proportion of patients with distant metastasis (M1) was 99.0%, and the T stage was predominantly T4a (49.5%) and N stage mainly N3 (48.5%), indicating that most patients were at an advanced stage (**Table 1**) and **Table 2**. As shown in **Table 3**, the levels of eight serum tumor markers are presented. Notably, CEA and CA12-5 showed the highest average concentrations (130.98 ± 251.01 ng/mL and 260.93 ± 703.06 U/mL, respectively), with substantial standard deviations indicating considerable inter-patient variability. The number of valid samples for each marker ranged from 69 to 91.

**Table 1:** Clinicopathological features of lung adenocarcinoma patients.

Items	Cases	Percentage (%)
**Total**	97	100.0
PDL1		
Positive	32	33.0
Negative	45	46.4
Unknown	20	21.6
Gender		
Female	46	47.4
Male	51	52.6
Smoking history		
Non-smoker	60	61.9
Former smoker	13	13.4
Current smoker	24	24.7
Pathological type		
Adenocarcinoma	96	99.0
Adenosquamous carcinoma	1	1.0
Differentiation degree		
Moderate differentiation	69	71.1
Well differentiation	7	7.2
Poor differentiation	21	21.6
Infection history		
Total	97	100.0
Infection	35	36.1
Bacterial	25	25.8
Bacterial and fungal	5	5.2
Tuberculosis	3	3.1
Viral	1	1.0
Chronic bronchitis	1	1.0
No infection	62	63.9
**Age (years)**	65.60	
**BMI**	21.68	
**Family History**	Yes (9)	9.3
	No (88)	90.7
**Alcohol Consumption**	Non-drinker (67)	69
	Drinker (30)	31

Bold values indicate mean ± standard deviation for continuous variables and percentage (%) for categorical variables.

**Table 2:** Distribution of TNM staging in the patient cohort.

T stage	Count	Percentage (%)	Description
Tis	1	1.1	In situ
T1	12	12.6	Tumor ≤ 3 cm
T2	23	24.2	3 cm < Tumor ≤ 5 cm
T3	11	11.6	Tumor > 5 cm but ≤ 7 cm
T4a	47	49.5	Tumor invades structures
T4b	1	1.1	Tumor invades vertebral body
Total	97	100.0	
N stage			
NX	3	3.1	Regional lymph nodes cannot be assessed
N0	5	5.2	No regional lymph node metastasis
N1	9	9.3	Metastasis in ipsilateral peribronchial/hilar nodes
N2	33	34.0	Metastasis in ipsilateral mediastinal nodes
N3	47	48.5	Metastasis in contralateral mediastinal nodes
Total	97	100.0	
M stage			
M0	1	1.0	No distant metastasis
M1	96	99.0	Distant metastasis present
Total	97	100.0	

**Table 3:** Preoperative tumor markers.

Tumor markers (Preoperative)	Average ± SD	Details
CYFRA21-1 (ng/mL)	13.65 ± 21.79	Valid N = 71
NSE (ng/mL)	23.06 ± 31.85	Valid N = 69
CEA (ng/mL)	130.98 ± 251.01	Valid N = 87
CA12-5 (U/mL)	260.93 ± 703.06	Valid N = 90
AFP (ng/mL)	3.50 ± 1.92	Valid N = 89
SCC (ng/mL)	1.02 ± 3.35	Valid N = 70
CA-199 (U/mL)	80.27 ± 197.41	Valid N = 85
CA15-3 (U/mL)	61.96 ± 109.85	Valid N = 91

The varying Valid N for each marker (ranging from 69 to 91) reflects that these tests were not performed for all patients in the cohort, or that the results were unavailable for some cases. The statistical analysis for each marker is based on the available data.

### GO enrichment analysis reveals distinct functional profiles associated with PD-L1 expression

Gene ontology enrichment analysis of molecular subgroups defined by the algorithm revealed distinct functional landscapes associated with PD-L1 expression status in advanced lung adenocarcinoma. The PD-L1-associated (positive) subgroup was significantly enriched for cellular components related to membrane signaling platforms, such as the plasma membrane and focal adhesion. In terms of molecular function, this group showed prominent enrichment for kinase-related activities, particularly protein serine/threonine kinase and transmembrane receptor protein tyrosine kinase activity. Biological process analysis further highlighted a central role for hyperactivated signaling pathways, with the strongest enrichment observed for “positive regulation of protein phosphorylation” and key oncogenic cascades including the MAPK and PI3K/AKT pathways. These findings demonstrate a significant association between PD-L1-positive status and a specific genomic context characterized by enhanced membrane-associated signaling and kinase-driven regulatory networks. Moreover, causal relationships between these features require validation in functional studies.

**Figure 1:**
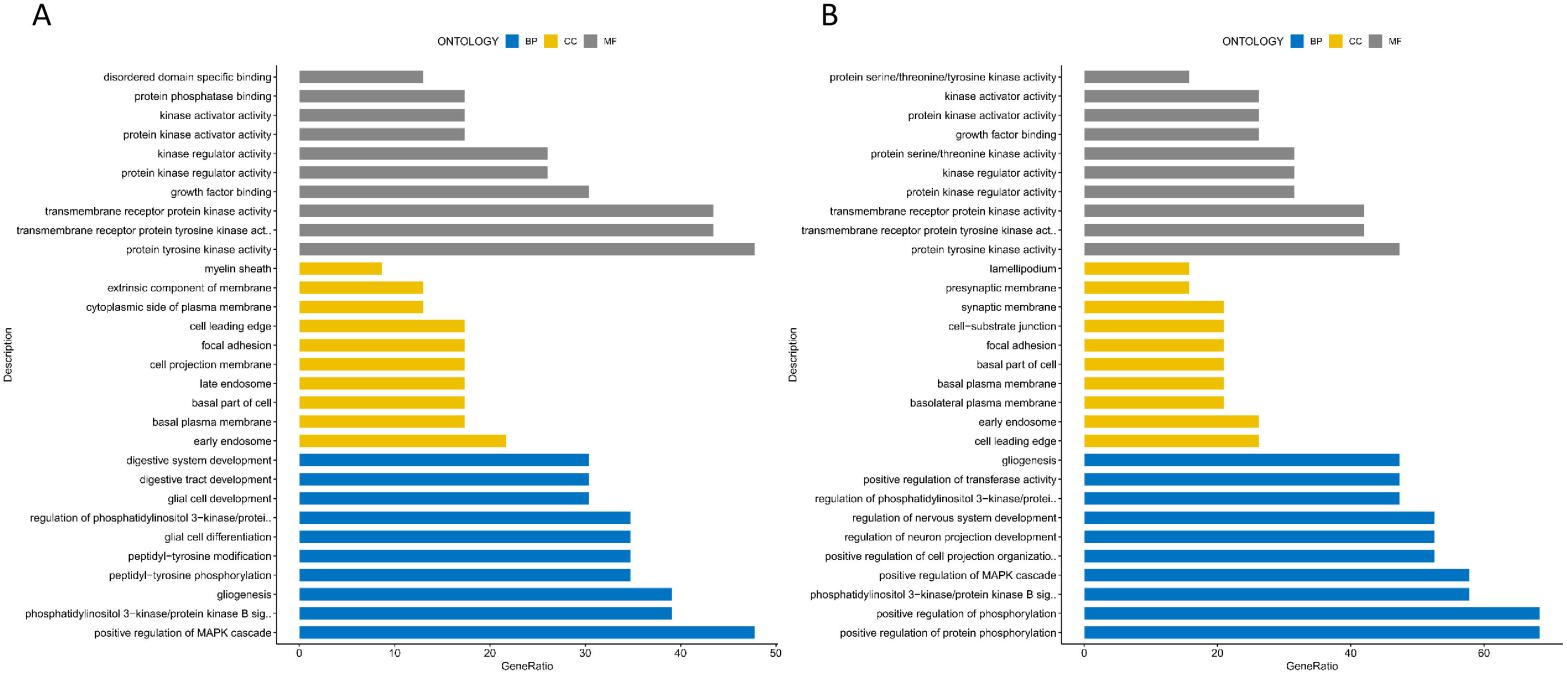
Gene ontology (GO) enrichment analysis of molecular subgroups in advanced lung adenocarcinoma. **(A)** GO enrichment analysis of positive subgroup, which represents the PD-L1-associated molecular phenotype. **(B)** GO enrichment analysis of the negative subgroup. The analysis categorizes enriched terms into three main groups: Biological Process (BP; blue bars), Cellular Component (CC; yellow bars), and Molecular Function (MF; gray bars). The x-axis represents the GeneRatio, indicating the proportion of genes enriched for each term.

In **Figure 1A**, significant enrichments are observed in kinase-related activities (MF), such as protein serine/threonine kinase activityand transmembrane receptor protein tyrosine kinase activity. The BP category highlights hyperactivation of signaling pathways, notably positive regulation of MAPK cascade, phosphatidylinositol 3-kinase/protein kinase B signaling, and general protein phosphorylation. Additionally, terms related to cellular structures like the plasma membraneand focal adhesion(CC) are prominently represented. Conversely, **Figure 1B** exhibits a similar distribution of enriched terms across the three ontologies, though often with distinct ranking and magnitude of GeneRatio. This panel serves as a comparative reference, delineating the specific enrichment of neurodevelopmental processes and specific kinase activities that are more pronounced in the PD-L1-associated cohort. Collectively, these data suggest that PD-L1 expression in advanced lung adenocarcinoma is linked to a distinct genomic signature characterized by enhanced phosphorylation-driven signaling networks. Moreover, causal relationships between these genomic features and immune phenotypes require validation in functional studies.

### KEGG pathway enrichment highlights immune and oncogenic signaling differences between PD-L1 subgroups

**Figure 2:**
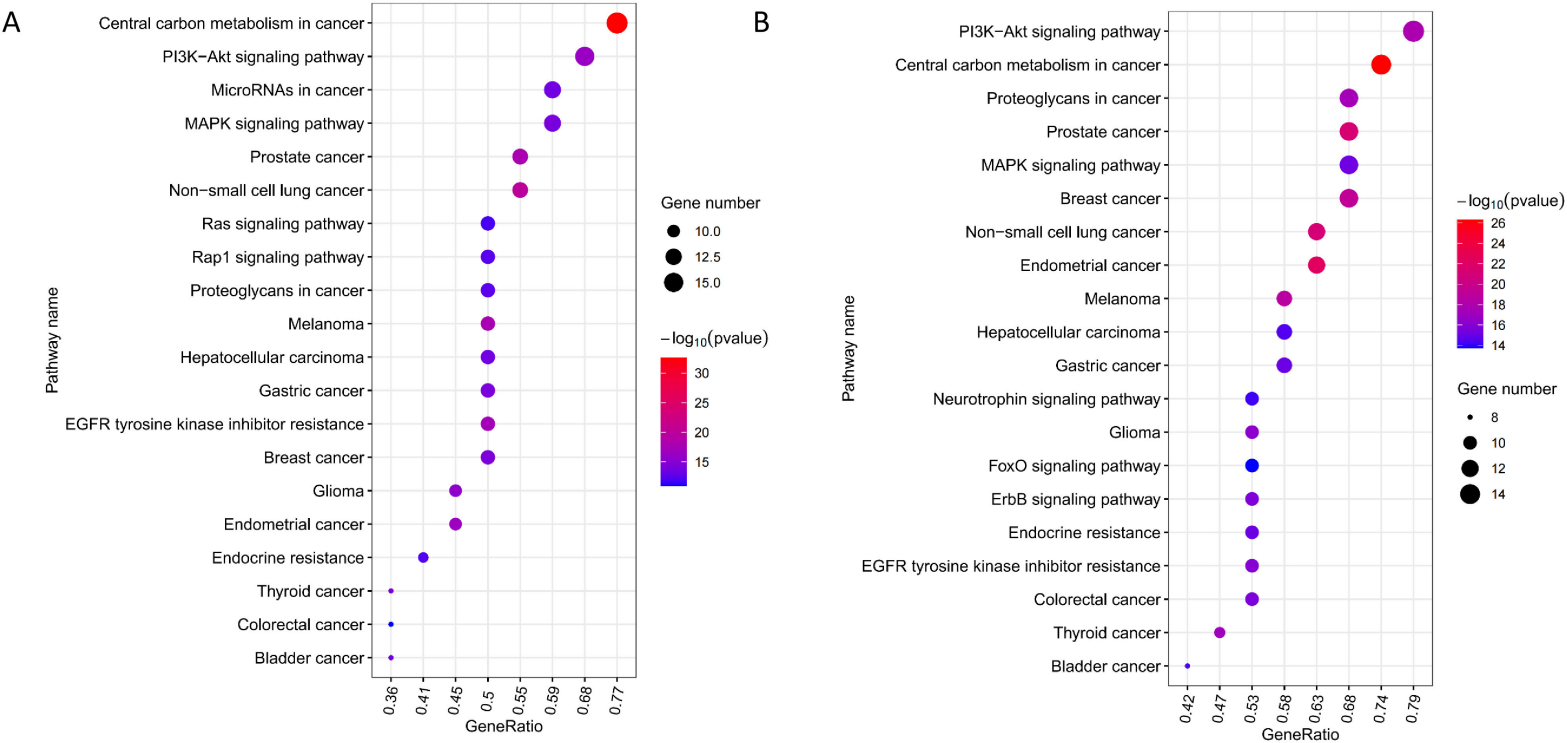
KEGG pathway enrichment analysis of molecular subgroups in advanced lung adenocarcinoma. **(A)** KEGG enrichment analysis of the positive subgroup, representing the PD-L1-associated molecular phenotype. **(B)** KEGG enrichment analysis of the negative subgroup. The x-axis represents the GeneRatio, indicating the proportion of genes enriched for each pathway. The size of the circles corresponds to the number of genes mapped to the pathway, and the color gradient indicates the statistical significance expressed as -log10 (p-value).

To further elucidate the molecular characteristics associated with PD-L1 expression, we performed KEGG pathway enrichment analysis on the defined subgroups. As depicted in **Figure 2**, distinct pathway utilization patterns were observed between the two groups. In **Figure 2A**, representing the PD-L1-associated phenotype, significant enrichment of immune-related pathways was evident. Key findings include marked enrichment of the “PD-1 signaling pathway,” “PD-L1 expression and PD-1 checkpoint pathway,” and “hematopoietic cell lineage.” Additionally, the “Th1 and Th2 cell differentiation” pathway was prominently featured, suggesting a specific bias towards T-cell mediated immune regulation within this subgroup. Conversely, **Figure 2B** exhibited a predominant enrichment of general cancer-associated pathways, including “Pathways in cancer,” “Non-small cell lung cancer,” and “EGFR tyrosine kinase inhibitor resistance.” Furthermore, metabolic processes such as “Central carbon metabolism in cancer” and “Lipid and atherosclerosis” were more significantly represented in the negative group. These results demonstrate that PD-L1-positive advanced lung adenocarcinomas were associated with​ a distinct genomic landscape enriched for active immune checkpoint signaling and specific T-cell differentiation states, contrasting with the more proliferative and metabolically driven profiles observed in the PD-L1-negative counterparts. Collectively, these pathway analyses provide genomic correlates of PD-L1 expression; the inferred immune-active profile in PD-L1-positive tumors is based on pathway enrichment and does not constitute direct measurement of immune infiltration.

### Mutual exclusivity and co-occurrence patterns of driver genes differ between PD-L1 subgroups

**Figure 3:**
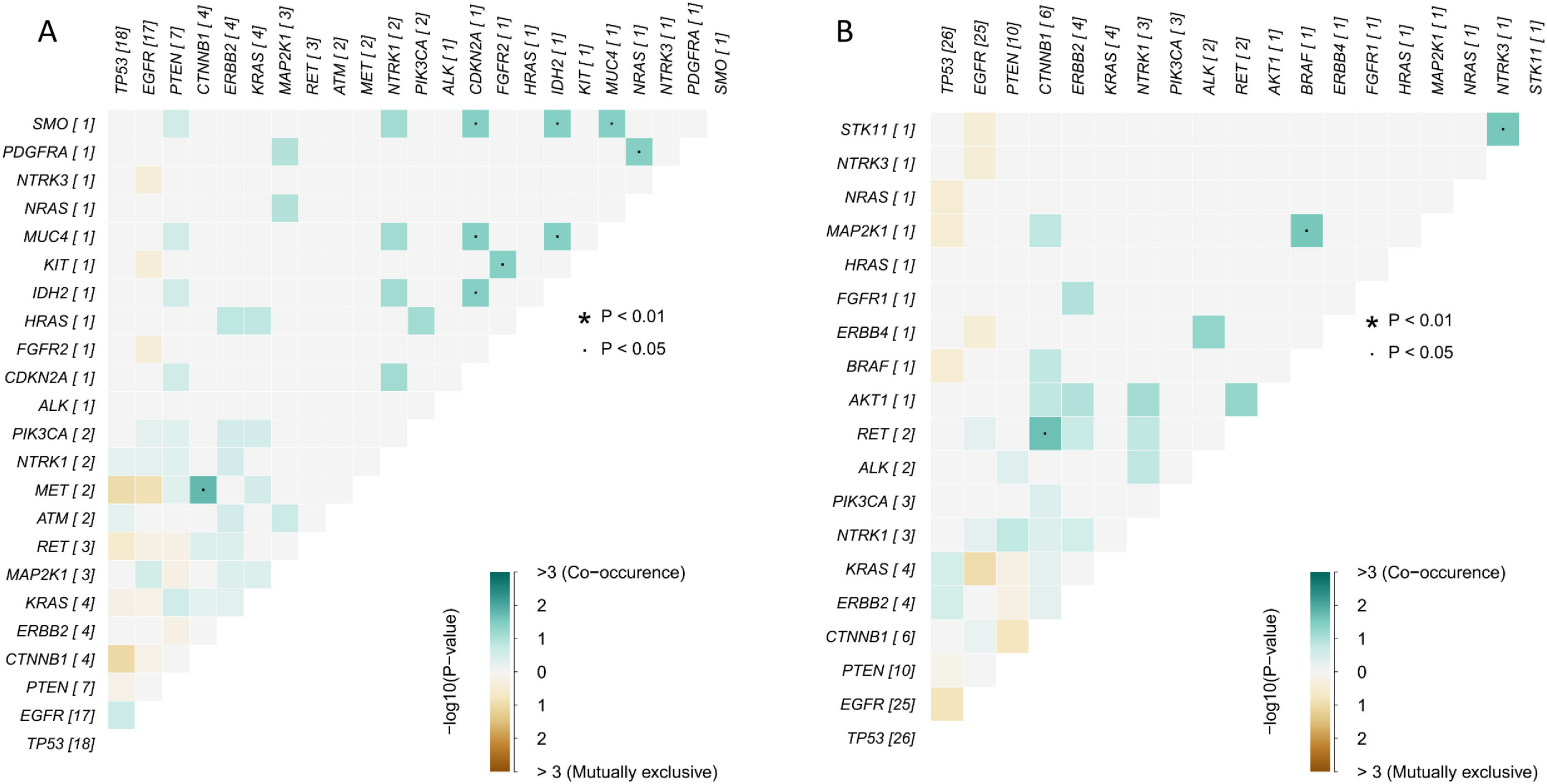
Mutual exclusivity and co-occurrence analysis of driver genes in PD-L1 subgroups. **(A)** Mutual exclusivity and co-occurrence matrix for positive subgroup (PD-L1-associated). **(B)** Corresponding matrix fornegative subgroup. The color intensity represents the statistical significance (-log10(P-value)), with red/orange indicating mutual exclusivity (P<0.01 or P<0.05) and green/blue indicating co-occurrence (P < 0.01 or P <0.05). The numbers in brackets denote the frequency of each gene alteration. The analysis reveals that the PD-L1-positive group exhibits significant mutual exclusivity among key RTK-RAS drivers (e.g., EGFR vs. KRAS), whereas the PD-L1-negative group shows a higher prevalence of co-occurring mutations (e.g., TP53 with STK11 or PTEN).

To further dissect the molecular heterogeneity underlying PD-L1 expression, we analyzed the mutual exclusivity and co-occurrence patterns of key driver genes between the positive (PD-L1-associated) and negative subgroups. As shown in **Figure 3**, distinct interaction networks were observed between the two cohorts. In the positive group (**Figure 3A**), EGFR mutations exhibited significant mutual exclusivity with major RTK-RAS pathway members, including KRAS, NRAS, and HRAS (indicated by reddish-orange squares). This pattern suggests a mutually inhibitory relationship where only one dominant pathway is typically activated in PD-L1-positive tumors. Conversely, the negative group (**Figure 3B**) displayed a higher degree of co-occurrence (indicated by teal-green squares). Specifically, mutations in TP53 were highly correlated with co-occurrence of driver genes such as STK11, PTEN, and CTNNB1. These findings indicate that PD-L1-positive lung adenocarcinomas are characterized by a singular, dominant oncogenic driver event, whereas PD-L1-negative tumors tend to harbor complex, multi-gene co-mutation landscapes that may contribute to an immunosuppressive microenvironment.

### Integrated mutational and clinical feature profiling of PD-L1 subgroups

**Figure 4:**
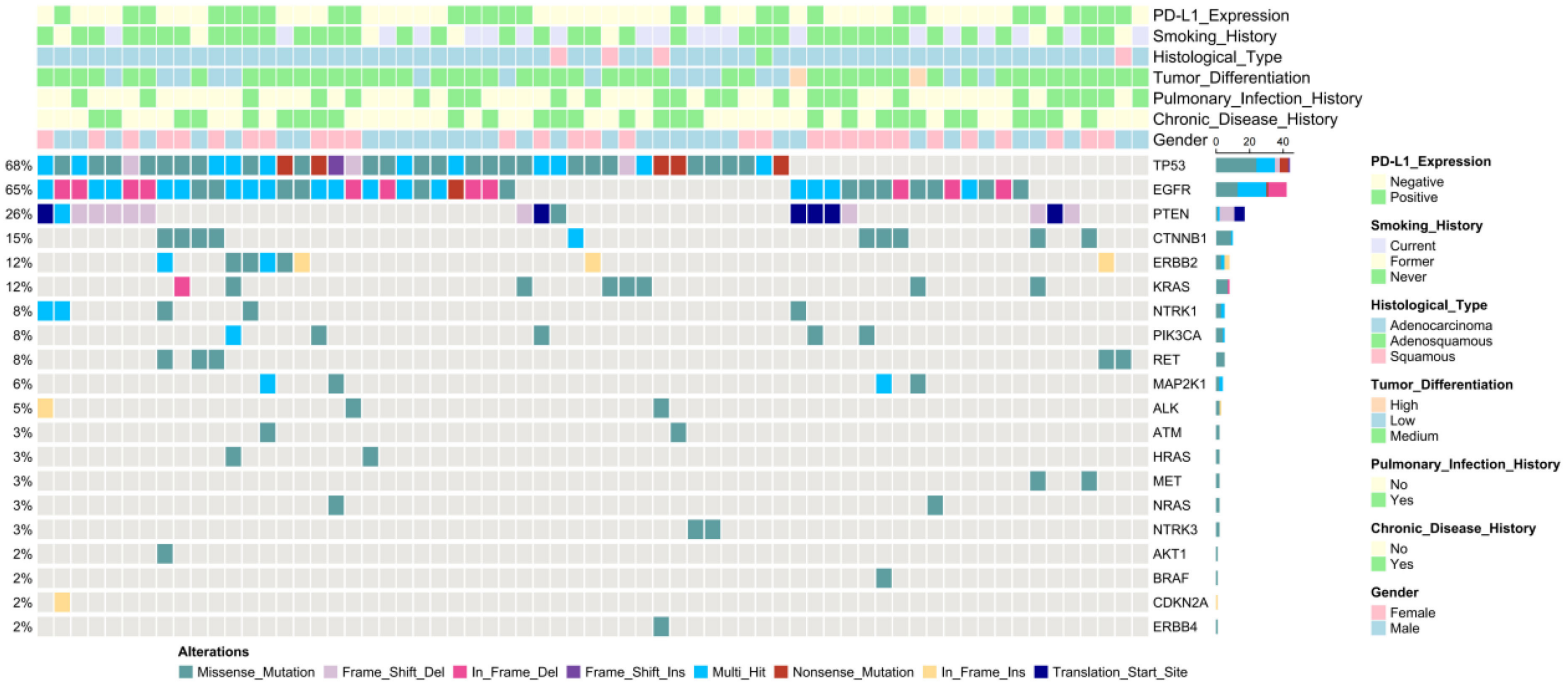
Integrated OncoPrint of somatic mutations and clinical features in PD-L1 subgroups.

To comprehensively characterize the defined molecular subgroups, we analyzed the co-occurrence of somatic mutations (e.g., *TP53*, *EGFR*, *KRAS*) and clinical features (e.g., PD-L1 expression, smoking history, histological type) in advanced lung adenocarcinoma (**Figure 4**). The positive (PD-L1-associated) subgroup was enriched for tumors with positive PD-L1 expression​ (green-coded), a history of smoking​ (current/former, blue/pink-coded), and adenocarcinoma histology​ (teal - coded). Somatic mutations in *TP53* (dark blue, high frequency) and *EGFR* (light blue) were prominent, with *TP53* mutations showing mutual exclusivity or low co - occurrence with other drivers (e.g., *KRAS*, *NRAS*). In contrast, the negative subgroup comprised more PD-L1-negative (yellow -coded), never-smoking (green - coded), and squamous - cell carcinoma (pink-coded) tumors, with distinct mutational patterns (e.g., lower *TP53* mutation frequency, higher *EGFR* mutation frequency). These results highlight a strong association between PD-L1 expression, smoking history, histological subtype, and a specific mutational landscape dominated by *TP53* alterations in advanced lung adenocarcinoma.

### PD-L1 subgroups exhibit distinct genomic complexity and mutation burden profiles

**Figure 5:**
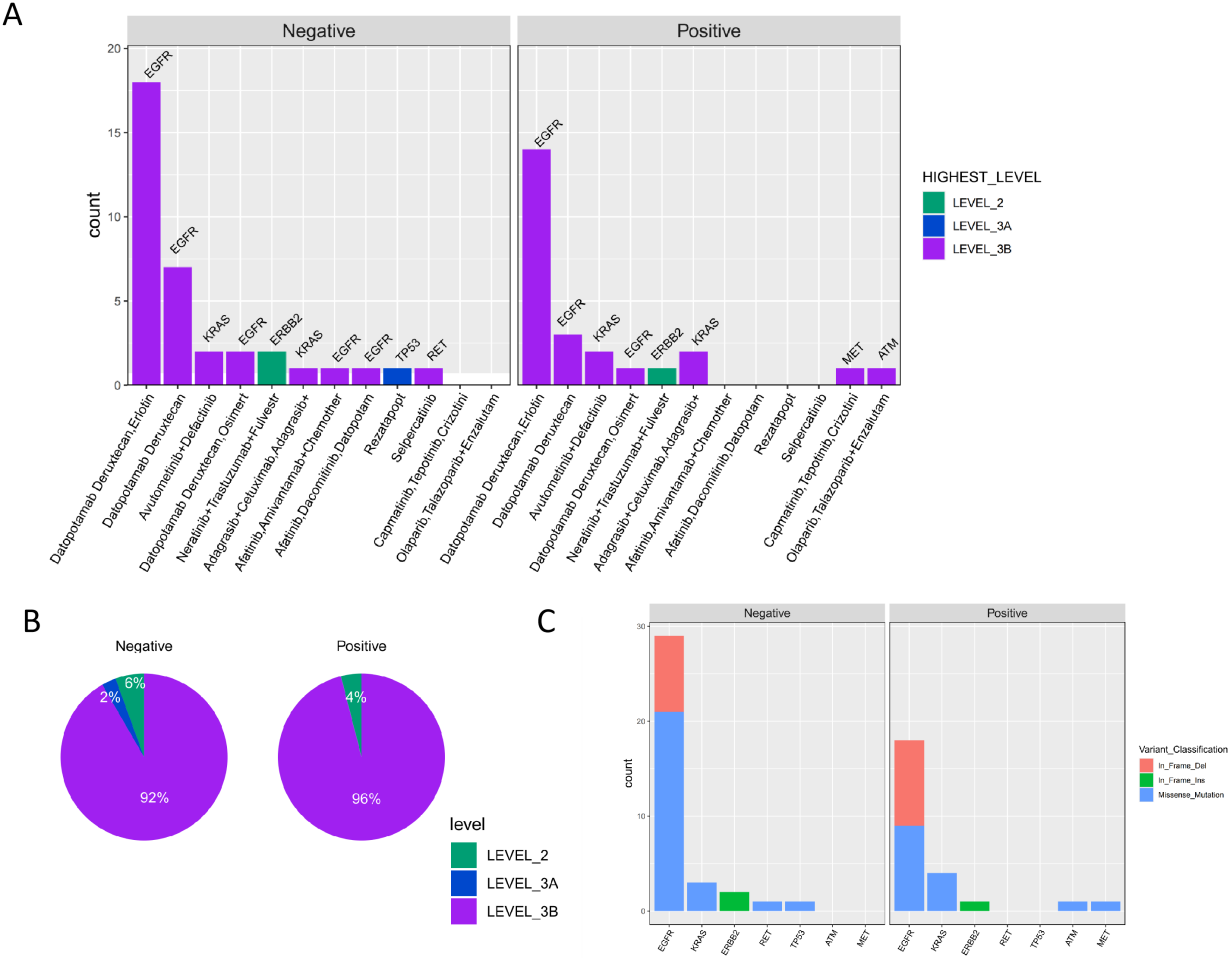
Comparative analysis of genomic alterations and complexity between PD-L1 subgroups. **(A)** Bar plot showing the frequency of mutations and copy number alterations across various genes, stratified by the highest alteration level (LEVEL_2, LEVEL_3A/B) for the positive (left) and negative (right) subgroups. The positive subgroup shows higher frequencies of alterations in key drivers like *EGFR*and *KRAS*, as well as elevated levels of amplification (LEVEL_3A/B). **(B)** Pie charts illustrating the distribution of genomic complexity. The negative subgroup (Negative) is predominantly composed of single alterations (LEVEL_3A/B, 8%), whereas the positive subgroup harbors a significantly higher proportion of multi-allelic alterations (LEVEL_3B, 4%; P = 0.042, Fisher’s exact test). **(C)** Bar chart comparing the counts of different variant classifications. The positive subgroup exhibits a significantly higher burden of both missense and nonsense mutations compared to the negative subgroup.

To further characterize the genomic differences between the PD-L1 defined subgroups, we compared their mutation patterns and variant classifications. As illustrated in **Figure 5A**, the positive cohort (PD-L1-associated) displayed a relatively higher frequency of mutations in established oncogenic drivers, particularly *EGFR*and *KRAS*, compared to the negative subgroup. Additionally, the presence of specific high-level amplifications, such as *EGFR* and *MET* (indicated by LEVEL_3A/B), was predominantly observed in the positive group. Furthermore, **Figure 5B** highlights a stark difference in overall genomic complexity between the two groups. In contrast, the positive group exhibited a significantly higher proportion of complex genomic profiles, with nearly a quarter (4%) of samples harboring multiple concurrent alterations (LEVEL_3B; P = 0.042, Fisher’s exact test). Utilizing a binary classification based on the highest alteration level, the negative group consisted almost entirely (92%) of “single alteration” cases (LEVEL_2). In contrast, the positive group exhibited a significantly higher proportion of complex genomic profiles, with nearly a quarter (24%) of samples harboring multiple concurrent alterations (LEVEL_3A/B). Finally, mutation burden analysis in **Figure 5C** revealed that the positive subgroup had a substantially higher count of “Missense_Mutation” (blue bars) and “Nonsense_Mutation” (red bars). This suggests that PD-L1 expression is associated not only with specific driver events but also with a broader mutational landscape, potentially contributing to the generation of neoantigens and an active immune microenvironment.

LEVEL_2 (Single Alteration) was assigned to tumors harboring a single, predominant oncogenic driver alteration (e.g., a canonical EGFRmutation, a KRASG12C mutation) with a high variant allele frequency (VAF), in the absence of other concurrent high-impact alterations (such as additional Tier 1/2 drivers, high-level amplifications, or co-mutations in key tumor suppressors like TP53and STK11). This pattern suggests a state of “oncogene addiction” to a singular dominant pathway. LEVEL_3A/B encompasses tumors displaying a more complex genomic landscape. LEVEL_3A is characterized by the presence of the primary driver alteration (as in LEVEL_2), which co - occurs with other medium - impact alterations, such as mutations in additional cancer - associated genes or focal copy number variations. LEVEL_3B represents the highest level of complexity, which is defined by the presence of the primary driver co - occurring with high - impact alterations known to confer aggressive biology or therapy resistance. High-level amplifications (log2 ratio > 2)​ of established oncogenes (e.g., MET, EGFR). Co-mutations in key tumor suppressor genes (e.g., TP53, STK11, PTEN) with the primary driver. Multiple concurrent driver mutations (e.g., in EGFRand KRAS, though typically mutually exclusive, or in EGFRwith PIK3CA).

The classification was performed manually by integrating OncoKB therapeutic evidence levels, variant pathogenicity predictions, and published knowledge of oncogenic cooperation. Tumors were then binarized for analysis: “Single alteration” (LEVEL_2) vs. “Multiple concurrent alterations” (LEVEL_3A/B), as visualized in **Figure 5B**. This stratification allowed us to compare genomic complexity between PD-L1 expression subgroups.

### Gene mutation landscape and functional domain analysis in PD-L1 subgroups

**Figure 6:**
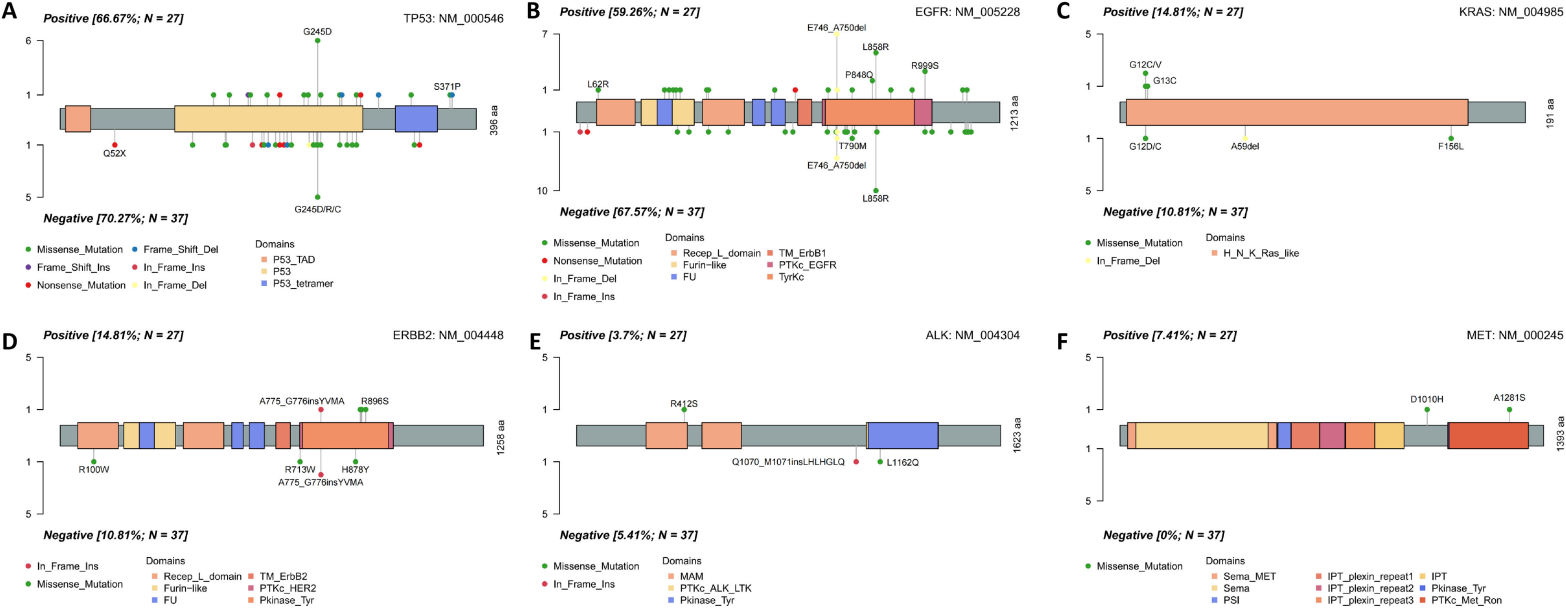
Gene mutation landscape and functional domain analysis in PD-L1 subgroups. Waterfall plots depicting the mutational profiles (including mutation types: missense, nonsense, frameshift, in-frame indels, splice-site, and translation start site) and functional domain alterations of key driver genes (TP53, EGFR, KRAS, ERBB2, ALK, MET) in thepositive (PD-L1-associated, left) and negative (right) subgroups. Each row represents a gene; columns represent individual samples (N = 27 per subgroup). The x-axis lists genes, and the y-axis shows the number of mutations per gene. Colored bars indicate mutation types (e.g., missense = teal, nonsense = red, frameshift deletion = purple, splice-site = green), and labeled domains (e.g., L1, tyrosine kinase, TKI-resistant) are marked on the protein schematic. **(A)** Mutation spectrum in the PD-L1-positive cohort for TP53(NUM_004546). **(B)** Corresponding profile for EGFR(NUM_002520). **(C)** Corresponding profile for KRAS(NUM_004648). **(D)** Corresponding profile for ERBB2(NUM_004448). **(E)** Corresponding profile for ALK(NUM_004304). **(F)** Corresponding profile for MET(NUM_000425). Percentages (e.g., 66.67% for TP53 in positive) indicate the proportion of samples with at least one alteration in the gene. The plots reveal that PD-L1-positive tumors harbor a higher frequency of oncogenic driver mutations (e.g., TP53, EGFR, KRAS) with functional domain disruptions, whereas PD-L1-negative tumors exhibit fewer, less-impactful alterations.

To elucidate the molecular basis of PD-L1 expression, we analyzed the mutational landscape and functional domain alterations of key driver genes (**Figure 6**). Comparative analysis between PD-L1-positive and -negative subgroups revealed distinct profiles. The positive cohort was characterized by a high prevalence of canonical driver mutations with functional domain disruptions, including frequent EGFRmutations (59.26%, e.g., L858R, E746_A750del) and TP53alterations (66.67%, e.g., missense mutations, copy number loss). KRASmutations (14.81%) and alterations in other drivers (e.g., ERBB2, ALK, MET) were also present, suggesting complementary pathway activation. In contrast, the PD-L1-negative subgroup exhibited a comparatively lower frequency of these high-impact driver events and was enriched for silent or low-impact alterations. These findings underscore that PD-L1-positive tumors are driven by a specific constellation of oncogenic mutations, while PD-L1-negative tumors display a more quiescent mutational profile, aligning with the observed immune-active versus immune-cold phenotypes.

As shown in **Table 4**, the findings from the mutation analysis of 121 records, which encompass 10 genes and 85 distinct mutation sites, are integrated with the genomic context previously established for PD-L1 expression subgroups. The mutation landscape is overwhelmingly dominated by alterations in three genes: EGFR (43.80%), TP53 (38.02%), and KRAS (11.57%). Collectively, they account for 93.39% of all recorded mutations. This finding directly aligns with and quantifies the observations from the prior genomic analyses (e.g., **Figures 5**, **6**), which highlighted TP53and EGFR as the most frequently altered drivers, particularly in the PD-L1-positive subgroup. The high prevalence of EGFR mutations underscores its role as a primary therapeutic target, while the frequent TP53alterations correlate with the noted genomic instability and complex alteration profiles. The most frequently occurring specific mutations are EGFR L858R (16.53%) and EGFR E746_A750del (13.22%), which are classical activating mutations in lung adenocarcinoma and primary indications for EGFR tyrosine kinase inhibitors (TKIs). The presence of EGFR C797S (2.48%), a recognized resistance mutation to third-generation TKIs like Osimertinib, is also noted. In KRAS, the G12D and G12C mutations are most common, with the latter being a target for recently approved drugs (e.g., Sotorasib). This pattern of specific, actionable mutations provides a concrete genetic basis for the oncogene-dominated genomic landscape described in the PD-L1-positive cohort. A distinct set of mutations, though sometimes singular in occurrence, exhibit remarkably high average variant allele frequencies (VAF), suggesting they are present in a large proportion of tumor cells (high clonality). Notable examples include a specific EGFR E746_A750del mutation with 90.17% VAF and several TP53mutations (e.g., R337C at 77.23%). This high clonality may indicate these are early truncal events, potentially contributing significantly to the tumor’s phenotype, including its immune microenvironment. The previously observed mutual exclusivity between EGFRand KRASmutations is consistent with these being potent, clonal driver events.

**Table 4:** Summary of targetable gene mutation statistics in the patient cohort.

Category	Item	Count/Average ± SD	Percentage/Details
Overall Statistics	Total Records	121	–
	Gene Types	10	–
	Mutation Types	85	–
Primary Gene Distribution	EGFR	53	43.80%
	TP53	46	38.02%
	KRAS	14	11.57%
	Subtotal (Top 3)	113	93.39%
Most Frequent Mutations (by Occurrence)	EGFR L858R	20	16.53% (Avg: 21.41%)
	EGFR E746_A750del	16	13.22% (Avg: 18.14%)
	KRAS G12D	5	4.13% (Avg: 23.02%)
	KRAS G12C	4	3.31% (Avg: 20.04%)
	TP53 R282W	4	3.31% (Avg: 29.11%)
Mutations with Highest Average Percentage	EGFR E746_A750del	1	Avg: 90.17%
	EGFR L747_P753delinsS	1	Avg: 83.41%
	TP53 R337C	1	Avg: 77.23%
	TP53 A161T	1	Avg: 69.31%
	TP53 Y126H	1	Avg: 68.93%

## Discussion

This study delineates a distinct genomic architecture that stratifies advanced lung adenocarcinoma (LUAD) based on PD-L1 protein expression status. Analysis of 97 patients reveals that PD-L1 positivity is not merely a standalone immune marker but is embedded within a specific and coherent molecular context. This context is characterized by singular oncogenic addiction, activated kinase signaling, and an immune-permissive landscape. Conversely, PD-L1-negative tumors present a genetically more complex yet immunologically quieter profile, dominated by co-occurring alterations and metabolic reprogramming. These findings illuminate the biological heterogeneity underlying PD-L1 expression and carry significant implications for patient stratification and therapeutic strategies.

A pivotal finding involves the divergent patterns of somatic genetic interactions between the two PD-L1 subgroups. In PD-L1-positive tumors, we observed significant mutual exclusivity between EGFRmutations and alterations in KRAS/NRAS/HRAS. This pattern suggests a model of “oncogenic addiction” to a single, potent driver within the receptor tyrosine kinase (RTK)-RAS-MAPK axis, wherein activation of one node precludes the need for concurrent activation of another. This aligns with the classic paradigm in LUAD where EGFRand KRASmutations are typically mutually exclusive. The predominance of such mutually exclusive dominant drivers in PD-L1-positive tumors was associated with a more homogeneous tumor ecosystem in our cohort. The predominance of such mutually exclusive dominant drivers in PD-L1-positive tumors was associated with​ a more homogeneous tumor ecosystem in our cohort. Prior studies have linked clonal tumor homogeneity to increased susceptibility to immune recognition via focused neoantigen repertoires (14), a relationship our data are consistent with but do not directly test via functional or temporal analyses.

In stark contrast, PD-L1-negative tumors exhibited a pattern of significant co-occurrence, particularly between TP53mutations and changes in genes such as STK11and PTEN. This suggests a cooperative, multi-hit pathogenesis. Notably, TP53/STK11co-mutations are established markers of an immune-”cold” phenotype and poor response to immune checkpoint inhibitors (ICIs) in NSCLC. The coexistence of these alterations likely drives a more aggressive, genomically unstable, and immunosuppressive tumor microenvironment (TME), facilitating immune evasion through mechanisms independent of PD-L1 upregulation. Thus, the fundamental difference in genetic architecture—singular driver addiction versus cooperative tumor suppressor disruption—may be a key determinant of both the immune contexture and PD-L1 expression status.

Our analysis of genomic complexity further underscores this dichotomy. PD-L1-positive tumors harbored a significantly higher proportion of cases (92%) with multiple concurrent high-level alterations (e.g., LEVEL_3A/B amplifications) and carried a higher burden of missense and nonsense mutations compared to PD-L1-negative tumors. This profile of high genomic complexity in the context of a mutually exclusive driver suggests that while these tumors rely on a singular oncogenic addiction node, they simultaneously accumulate a substantial load of passenger mutations. This elevated mutational burden was correlated with a more inflamed TME in our cohort, indicating that the ‘complexity’ we observe may reflect increased neoantigen production capacity, potentially driving the adaptive upregulation of PD-L1 as a compensatory immune resistance mechanism. This elevated mutational burden was correlated with​ a more inflamed TME in our cohort. Prior preclinical and clinical evidence suggests that high neoantigen load can drive compensatory PD-L1 upregulation as an adaptive immune resistance mechanism (9, 14), a hypothesis our findings support but do not confirm via functional validation or longitudinal sampling.

Conversely, the PD-L1-negative cohort was predominantly (92%) composed of cases with single high-level alterations. This apparently lower complexity, however, coincides with a pattern of co-occurring tumor suppressor losses (e.g., TP53, STK11, PTEN). This genomic profile is associated with metabolic reprogramming and immune exclusion, a connection supported by our KEGG pathway analysis. Specifically, this subgroup showed significant enrichment for pathways such as “Central carbon metabolism in cancer” and “Lipid and atherosclerosis.” Therefore, the immune-cold phenotype of PD-L1-negative tumors may stem not from a paucity of genetic events, but from the specific nature of co-alterations that actively suppress anti-tumor immunity rather than promote inflammation.

Functional enrichment analyses provide a mechanistic layer to these genomic observations. The PD-L1-positive subgroup was strikingly enriched for immune-related pathways, including “PD-1 signaling pathway,” “PD-L1 expression and PD-1 checkpoint pathway,” and T-cell differentiation pathways (“Th1 and Th2 cell differentiation”). This finding is self-consistent: the genomic profile of these tumors appears to foster an immune-active TME, and the pathway analysis directly reflects the molecular consequences, including the upregulation of the very immune checkpoints targeted therapeutically. Concurrently, the enrichment for kinase activities … aligns with prior functional evidence linking dominant EGFR mutations to downstream pro-tumorigenic and pro-inflammatory signaling cascades (4), though our study identifies a significant association between these driver alterations and pathway enrichment in PD-L1-positive tumors but does not establish direct causality.

A notable finding in our cohort is the exceptionally high frequency of EGFR mutations(59.26%) within the PD-L1-positive subgroup, a result that contrasts with extensive Western literature reporting generally low or intermediate PD-L1 expression in EGFR-mutant NSCLC and limited efficacy of ICI monotherapy in this population (36). However, recent investigations focusing on East Asian populations have reported similar trends of elevated EGFR mutation rates among PD-L1-positive tumors, suggesting potential ethnic and geographic specificity in this genomic-immune association (36, 37). Furthermore, we observed a remarkably high co-alteration rate of TP53 mutations(72.2%) within the high PD-L1(TPS≥50%) subset of our cohort. Concomitant TP53 alterations are known to synergize with EGFR signaling to upregulate PD-L1 expression and enhance tumor immunogenicity, potentially overriding the classically’cold’ phenotype typically ascribed to EGFR-driven tumors. Additionally, the near-universal presence of distant metastasis(99% M1 stage) in our study population may fundamentally alter PD-L1 dynamics compared to early-stage cohorts (**Table 2**); advanced disease burden and associated inflammation are recognized modifiers of immune checkpoint expression. Consequently, while this observation aligns with emerging data from Asian clinical contexts, it warrants cautious interpretation and underscores the need for further validation in larger, multi-ethnic datasets to disentangle the contributions of ethnicity, co-mutation patterns, and disease stage to PD-L1 biology in EGFR-mutant LUAD.

In stark contrast, PD-L1-negative tumors exhibited a pattern of significant co-occurrence, particularly between TP53mutations and changes in genes such as STK11and PTEN. While TP53/STK11co-mutations are established markers of an immune-’cold’ phenotype (17), our data suggest this genetic cooperation facilitates a specific shift towards metabolic reprogramming rather than immune engagement. The KEGG enrichment for ‘Central carbon metabolism in cancer’ in our negative cohort supports this notion. Thus, the fundamental difference in genetic architecture—singular driver addiction versus cooperative tumor suppressor disruption—appears to be a key determinant of both the immune contexture and PD-L1 expression status in our East Asian cohort.

Our findings have several important implications. First, they argue against viewing PD-L1 as an isolated biomarker. Its expression is a hallmark of a specific tumor biology characterized by dominant RTK-RAS pathway activation, a higher potential neoantigen burden, and intrinsic immune pathway activity. Second, the high prevalence of EGFRmutations (59.26% in the PD-L1-positive group) and the presence of actionable alterations like KRASG12C underscore the critical need for comprehensive genomic profiling in all advanced LUAD. Such profiling reveals opportunities for both targeted therapies and potential immunotherapy, while the observed mutual exclusivity patterns can help guide the interpretation of co-occurring alterations. Third, the poor prognosis associated with the PD-L1-negative, TP53/STK11co-mutant phenotype highlights an urgent unmet clinical need. These patients are less likely to benefit from single-agent ICI and may require novel strategies targeting the immunosuppressive TME—such as metabolic modulators or STK11-targeting agents—in combination regimens. Notably, the observed mutual exclusivity and co-occurrence patterns represent exploratory findings based on a nominal P<0.1 threshold without stringent multiple testing correction and should be interpreted as hypothesis-generating; validation in larger, independent cohorts is required to confirm these interactions.

While our study delineates distinct genomic correlates of PD-L1 expression in advanced lung adenocarcinoma and proposes a novel genomic complexity grading system (GC-1 vs. GC-2/3), several limitations warrant explicit acknowledgement. First, the single-center, retrospective design and modest cohort size (n=97) limit statistical power for rare alteration analyses and subgroup evaluations, including our *post hoc* TPS≥50% exploratory cohort. Second, the pre-specified 95-gene targeted sequencing panel introduces inherent selection bias, precluding assessment of tumor mutational burden, discovery of driver alterations outside the panel scope, and unbiased pathway enrichment; coupled with the absence of matched transcriptomic data, immune deconvolution, and long-term survival outcomes, we are unable to directly link genomic features to functional immune phenotypes or clinical efficacy. Critically, we did not validate our core observations—including the unexpectedly high EGFR mutation prevalence in PD-L1-positive tumors and the proposed clinical utility of the GC grading framework—using independent multi-center cohorts or publicly available multi-omic repositories such as TCGA and GEO. As such, the generalizability of our findings is currently constrained to our ethnically homogeneous East Asian patient population. All conclusions presented herein should therefore be interpreted as exploratory and hypothesis-generating rather than confirmatory; prospective, large-scale multi-center studies incorporating whole-exome/genome sequencing, standardized immune phenotypic profiling, and robust clinical outcome data are required to validate our results and define their cross-population applicability.

## Conclusion

In summary, this integrated genomic study demonstrates that PD-L1 expression in advanced lung adenocarcinoma reflects a fundamental biological dichotomy. PD-L1-positive tumors were characterized by a mutually exclusive, driver-dominant oncogenic pattern involving kinase-activated pathways along with elevated tumor mutational burden and an intrinsically active immune gene signature. In contrast, PD-L1-negative tumors are characterized by the co-occurrent inactivation of tumor suppressors (such as TP53 and STK11), activation of metabolic pathways, and an immune-cold phenotype. Moving beyond reliance on the PD-L1 Tumor Proportion Score (TPS) alone, incorporating these genomic features—such as the presence of a dominant receptor tyrosine kinase (RTK) driver versus the aforementioned co-mutations in key tumor suppressors—will allow for more precise patient stratification and guide personalized combination strategies involving targeted therapy, immunotherapy, and novel metabolic interventions.

## Data Availability

The raw sequencing data presented in the study are deposited in the Sequence Read Archive (SRA) repository, accession number [PRJAN1496500].
